# A nation-wide twin study of social cognition in schizophrenia spectrum disorders

**DOI:** 10.1038/s41537-022-00223-1

**Published:** 2022-03-02

**Authors:** Cecilie K. Lemvigh, Birte Y. Glenthøj, Birgitte Fagerlund

**Affiliations:** 1 Center for Clinical Intervention and Neuropsychiatric Schizophrenia Research (CINS) and Center for Neuropsychiatric Schizophrenia Research (CNSR), Mental Health Center, Glostrup, Copenhagen University Hospital – Mental Health Services CPH, Copenhagen, Denmark; 2 Department of Clinical Medicine, Faculty of Health and Medical Sciences, University of Copenhagen, Copenhagen, Denmark; 3 Department of Psychology, Faculty of Social Sciences, University of Copenhagen, Copenhagen, Denmark

**Keywords:** Psychosis, Neuroscience

## Abstract

We examined social cognition in 32 monozygotic (MZ) and 21 dizygotic (DZ) twin pairs concordant or discordant for a schizophrenia spectrum diagnosis and healthy control (HC) twin pairs (29 MZ/20 DZ). All participants were recruited through the Danish registers. Patients showed several deficits in the ability to detect sarcasm. Impairments were also observed in the unaffected MZ co-twins, indicating that social cognitive deficits could be a genetic vulnerability indicator of the disease. Worse social cognition was associated with lower intelligence and higher levels of psychopathology in patients.

## Introduction

Social cognitive deficits are a well-established finding in patients with schizophrenia and these deficits are strongly related to functional outcome^1^. One example of a social cognitive function that is impaired in patients with schizophrenia is the ability to detect sarcasm^2,3^. The ability to detect sarcasm requires intact theory of mind^4,5^ as well as social perception^6^, i.e. the processes involved in making inferences about complex/ambiguous social situations using verbal or non-verbal cues, with impairments resulting in misinterpretations of the intent of others^7^. Social cognition is also impaired in first-degree relatives of patients with schizophrenia, suggesting that these deficits may be related to the genetic vulnerability of the disorder^8^. Previous twin studies have established that most cognitive functions are strongly influenced by genetics^9^ and show genetic overlap with schizophrenia liability, indicating that shared genetic factors influence cognition and schizophrenia risk^10–12^. However, twin studies of social cognition in schizophrenia are lacking^9,13^.

## Results

### Group differences in TASIT

**Fig. 1:**
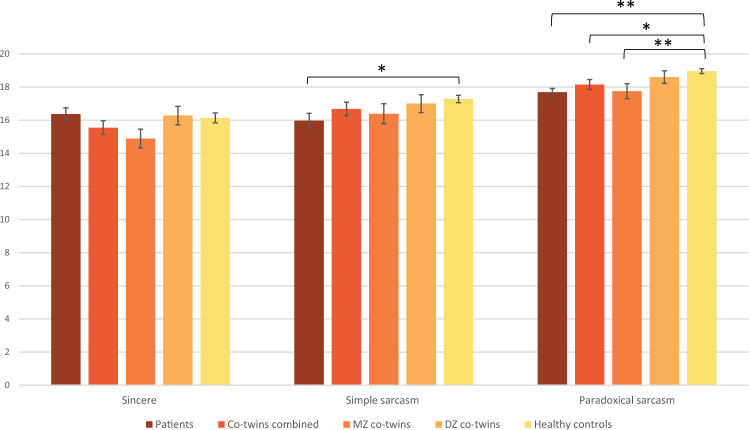
Average TASIT performance. TASIT performance for patients, their unaffected co-twins (combined and split on zygosity) and healthy control twin pairs showing the average number of correct responses. Error bars represent the standard error of the mean (SEM). *significant at *p* = 0.05, ** Significant after FDR corrections.

Figure 1 shows the average performance of patients, unaffected co-twins and HCs in TASIT. There were no group differences between patients and HCs in the sincere condition, *U* = 2853.5, *p* = 0.515, *r* = −0.05. Patients performed worse than HCs in both the simple, *U* = 2378.5, *p* = 0.020, *r* = −0.18, and paradoxical sarcastic conditions, *U* = 2073.0, *p* < 0.001, *r* = −0.28, although only the difference in paradoxical sarcasm remained significant after correction for multiple comparisons. The unaffected co-twins performed similar to HCs in the sincere, *U* = 2252.5, *p* = 0.176, *r* = −0.11, and simple sarcastic conditions, *U* = 2384.0, *p* = 0.401, *r* = −0.07, while a significant group difference was observed in the paradoxical sarcastic condition, *U* = 2040.5, *p* = 0.022, *r* = −0.17, but this did not survive FDR corrections. When the unaffected co-twin group was split according to zygosity, only MZ co-twins performed worse than HCs in the paradoxical sarcastic condition, *U* = 927.0, *p* = 0.006, *r* = −0.25, while DZ co-twins performed similar to controls, *U* = 1113.5, *p* = 0.452, *r* = −0.07. This finding survived correction. Finally, there were no significant differences between patients and their unaffected co-twins within discordant proband pairs in either condition (sincere: *Z* = −1.646, *p* = 0.100, *r* = −0.17, simple sarcasm: *Z* = −0.533, *p* = 0.594, *r* = −0.05, paradoxical sarcasm: *Z* = −0.755, *p* = 0.450, *r* = −0.08).

### Associations with IQ, psychopathology and functioning

There were no associations between DART and TASIT, but performance in the paradoxical condition correlated with both vocabulary and block design in the whole sample. When the sample was split into the three groups, the correlations with block design and vocabulary remained in the patients, but only the association with block design survived FDR corrections. In the unaffected co-twin group, paradoxical sarcasm also correlated with block design and vocabulary, although none of these survived corrections. No correlations between TASIT and measures of IQ were observed in the HCs. In patients, simple sarcasm correlated moderately with negative and general symptoms from the PANSS, while paradoxical sarcasm correlated moderately with positive, negative and general symptoms. No associations between TASIT and PANSS were evident in the unaffected co-twins or HCs. Finally, TASIT performance correlated with GAF in patients and unaffected co-twins, although only the findings in the patient group survived corrections for multiple comparisons (Table 1).

**Table 1:** Inter-correlations between social cognition and measures of IQ, psychopathology and level of functioning.

	DART	Vocabulary	Block design	PANSS positive	PANSS negative	PANSS general	GAF
Whole sample (*N* = 213)							
TASIT sincere	0.036 (*p* = 0.612)	0.026 (*p* = 0.709)	0.096 (*p* = 0.161)	0.005 (*p* = 0.941)	−0.011 (*p* = 0.875)	0.026 (*p* = 0.709)	0.031 (*p* = 0.663)
TASIT simple sarcasm	−0.073 (*p* = 0.300)	−0.044 (*p* = 0.527)	0.69 (*p* = 0.319)	−0.028 (*p* = 0.691)	−0.057 (*p* = 0.410)	−0.048 (*p* = 0.488)	0.029 (*p* = 0.684)
TASIT paradoxical sarcasm	0.096 (*p* = 0.174)	0.242** (*p* < 0.001)	0.300** (*p* < 0.001)	−0.307** (*p* < 0.001)	−0.311** (*p* < 0.001)	−0.321** (*p* < 0.001)	0.362** (*p* < 0.001)
Patients (*N* = 62)							
TASIT sincere	−0.19 (*p* = 0.885)	0.108 (*p* = 0.403)	0.253** (*p* = 0.047)	−0.157 (*p* = 0.223)	−0.340** (*p* = 0.007)	−0.329** (*p* = 0.009)	0.428** (*p* = 0.001)
TASIT simple sarcasm	0.074 (*p* = 0.575)	0.096 (*p* = 0.456)	0.148 (*p* = 0.252)	−0.013 (*p* = 0.923)	0.078 (*p* = 0.549)	0.152 (*p* = 0.239)	−0.135 (*p* = 0.310)
TASIT paradoxical sarcasm	0.054 (*p* = 0.687)	0.327* (*p* = 0.009)	0.371** (*p* = 0.003)	−0.366** (*p* = 0.003)	−0.356** (*p* = 0.005)	−0.305** (*p* = 0.016)	0.334** (*p* = 0.010)
Unaffected co-twins (*N* = 53)							
TASIT sincere	0.132 (*p* = 0.365)	−0.175 (*p* = 0.209)	−0.162 (*p* = 0.248)	0.167 (*p* = 0.236)	0.181 (*p* = 0.200)	0.034 (*p* = 0.810)	−0.151 (*p* = 0.289)
TASIT simple sarcasm	−0.180 (*p* = 0.215)	−0.159 (*p* = 0.254)	−0.022 (*p* = 0.878)	0.214 (*p* = 0.128)	0.058 (*p* = 0.685)	0.223 (*p* = 0.112)	−0.260 (*p* = 0.065)
TASIT paradoxical sarcasm	0.142 (*p* = 0.329)	0.320* (*p* = 0.020)	0.332* (*p* = 0.015)	−0.194 (*p* = 0.167)	−0.188 (*p* = 0.182)	−0.262 (*p* = 0.060)	0.322* (*p* = 0.021)
Healthy controls (*N* = 98)							
TASIT sincere	0.035 (*p* = 0.736)	0.078 (*p* = 0.448)	0.198 (*p* = 0.051)	−0.056 (*p* = 0.587)	0.001 (*p* = 0.991)	0.160 (*p* = 0.118)	0.029 (*p* = 0.783)
TASIT simple sarcasm	−0.100 (*p* = 0.329)	−0.119 (*p* = 0.245)	0.013 (*p* = 0.903)	0.149 (*p* = 0.146)	0.004 (*p* = 0.968)	0.018 (*p* = 0.863)	−0.057 (*p* = 0.591)
TASIT paradoxical sarcasm	0.168 (*p* = 0.101)	0.101 (*p* = 0.324)	0.110 (*p* = 0.282)	−0.110 (*p* = 0.282)	−0.154 (*p* = 0.133)	−0.104 (*p* = 0.310)	0.194 (*p* = 0.065)

Values represent spearman’s correlations.

*DART* Danish version of the National Adult Reading test, *PANSS* The Positive and Negative Syndrome Scale, *GAF* Global Assessment of Functioning.

*Significance level under *p* = 0.05.

**Significant after corrections for multiple comparisons.

## Discussion

Patients with schizophrenia spectrum disorders showed impairments in the detection of both simple and paradoxical sarcasm, with small to moderate effect sizes, in line with the previous findings^2,3^. Unaffected co-twins performed worse than controls on paradoxical sarcasm only, although the effect was small. When the group was split on zygosity, only MZ co-twins differed from controls, suggesting that impaired understanding of paradoxical sarcasm may be related to the genetic vulnerability of schizophrenia spectrum disorders.

The finding that the ability to understand sarcastic interactions was moderately associated with measures of current IQ is also in line with the previous literature^2^, although other evidence suggests that social cognition represents a distinct domain separate from non-social cognition^14^. These associations were not evident in the HCs, indicating that social cognition in patients (and to some extent in unaffected co-twins) may in part depend on general cognitive abilities, whereas social cognition in normal development may represent a specialised function. However, this finding should be interpreted with caution due to ceiling effects in TASIT performance in the HCs and should be explored further in future studies.

In addition, the observed impairments in the understanding of paradoxical sarcasm correlated moderately with measures of psychopathology in the patients. This is consistent with some previous studies, although the literature regarding clinical correlates of social cognitive deficits in schizophrenia is mixed^2,15–18^. Exploratory analyses (not corrected for multiple comparisons) revealed that the correlations with positive symptoms were driven by items covering delusions, hallucinatory behaviour, excitement, suspiciousness/persecution (strongest association) and hostility; Negative symptoms were due to associations with blunted affect, poor rapport and difficulty in abstract thinking (strongest association). Finally, associations with general symptoms were driven by mannerisms/posturing, motor retardation, poor attention, disturbances of volition and poor impulse control. Impairments in the ability to detect sarcasm were also related to the level of functioning in the patient group, and taken together, these findings suggest that this aspect of social cognition may represent a relevant target for treatment efforts.

A major strength of the current study is the use of a twin design, which holds considerable advantages compared to studies of first-degree relatives^19^. The inclusion of twin pairs allows for an examination of the graded genetic proximity and may potentially limit the effects of early environmental influences, as the twins are born at the same time and in most cases raised under similar conditions. Limitations include ceiling effects observed in TASIT performance and inadequate variance making these data unsuitable for genetic twin modelling. Moreover, although we were able to identify all twin pairs nationwide through the Danish registers, the scarcity of twins with a schizophrenia spectrum disorder in combination with the fact that this patient group is typically difficult to recruit, another potential limitation concern the number of participants included in the study which raises concerns about power issues. Finally, we only examined a very narrow subcomponent of social cognition and other measures of social cognition may be closer related to real-world functioning^20^. Nevertheless, TASIT has demonstrated good psychometric properties, including acceptable test-retest reliability and internal consistency^20^. More twin studies of social cognition are needed, including examinations of other domains of social cognition^7^. Previous twin studies of cognition have suggested that the basic genetic architecture of schizophrenia shows overlap with cognition^10–12^, and therefore future studies should examine potential genetic associations between social cognition and schizophrenia liability and how this cognitive domain relates to IQ to further understand the genetic underpinnings of social cognitive deficits in schizophrenia. A better understanding of the pathophysiological processes underlying cognitive impairments in schizophrenia spectrum disorders may increase our understanding of the aetiology of the illness leading from genes to psychopathology.

## Methods

### Participants

The study was approved by The Danish Health and Medicines Authority, The Danish National Committee on Health Research Ethics (H-2-2010-128), and The Danish Data Protection Agency (2010-41-5468) and written informed consent was obtained from all participants. We recruited monozygotic (MZ) and dizygotic (DZ) twin pairs concordant or discordant for a schizophrenia spectrum diagnosis (proband pairs) as well as healthy control (HC) twin pairs through the Danish registers to minimise ascertainment bias. Inclusion criteria included: Age 18–60 years and both twins alive and residing in Denmark. Exclusion criteria included: Serious head trauma or physical illness, pregnancy, and a diagnosis of drug/alcohol addiction. A further exclusion criterium for HC pairs was major psychosis in first-degree relatives. HCs were matched on age and gender to the included proband pairs. In total, 213 twins (Mean age = 40.7, SD = 10.4; 48.6% females) participated in this study (32 complete MZ proband pairs, 21 complete DZ proband pairs, 29 complete MZ HC pairs, 20 complete DZ HC pairs). Additionally, nine twins participated without their siblings.

### Assessments

Register diagnoses were verified using the Schedules for Clinical Assessment in Neuropsychiatry (SCAN) interview according to ICD-10 criteria^21^. Diagnoses included schizophrenia (N = 37), schizotypal disorder (*N* = 11), acute and transient psychotic disorders (*N* = 9), schizoaffective disorders (*N* = 4) and unspecified nonorganic psychosis (*N* = 1). The majority of the proband pairs were discordant for the disorder (Further details about the cohort can be found in refs. ^11,12^).

The Danish version of The Awareness of Social Inferences Test (TASIT) – Part A2 Social Inference (minimal) was used to examine the ability to detect sarcasm^22^. The Danish version of the National Adult Reading Test (DART) was used to estimate premorbid intelligence (IQ)^23^, while two subtests (vocabulary & block design) from the Wechsler Adult Intelligence Scale—Third edition were used as measures of current IQ^24^ (Please see Table 2 for brief descriptions of the tasks). The Positive and Negative Syndrome Scale (PANSS) was used to assess psychopathology^25^. We also included the Global Assessment of Functioning Scale (GAF)^26^.

**Table 2:** Descriptions of the included cognitive tasks.

Test	Description
**TASIT**	The test is comprised of 15 small video clips of professional actors performing everyday interactions representing conversational exchanges that typically occur between a couple, friends, or colleagues. The clips last between 15 and 50 s. The task is to figure out if the actors are being sincere or sarcastic based on the tone of voice, facial expressions, gestures and body postures. There are three conditions; Sincere (five clips), simple sarcasm (five clips), and paradoxical sarcasm (five clips). In the *sincere* condition, there is congruence between what the actor is literally saying and what is meant, i.e. the verbal message and the context are consistent. Contrary, in the sarcastic conditions, there is an incongruence between the spoken words and the paralinguistic and facial cues. The *simple sarcasm* can only be recognised by interpreting these cues correctly and identifying the contradictions. In the *paradoxical sarcasm* conditions, the dialogue only makes sense if the sarcasm is recognised, i.e. a literal interpretation of the interaction is meaningless. After viewing a clip, the participant is asked four questions pertaining to the intentions of the actors (what they were doing, saying, thinking, and feeling) with a max score of 4 points per clip resulting in a total score of max 20 for each of the three conditions.
**DART**	Participants are asked to read aloud a list of 50 words with irregular spelling
**WAIS-III** Vocabulary Block design	Participants are asked to explain the meaning of/define a list of words Participants are asked use red-and-white blocks to construct specific patterns according to a picture

### Statistical analyses

Statistical analyses were performed using SPSS (version 25.0, SPSS Inc.). The TASIT data were not normally distributed and non-parametric two-tailed tests were applied. Group differences between patients and HCs as well as between unaffected co-twins and HCs were examined using the Mann–Whitney U-test. Group differences between patients and their unaffected co-twins were examined using the related samples Wilcoxon signed-rank test. Effect sizes were calculated as $r = Z/\surd N$, with r values of 0.1, 0.3 and 0.5 indicative of small, medium and large effects respectively^27^. Associations between TASIT and measures of IQ/psychopathology were examined using Spearman’s correlations. Results were corrected for multiple comparisons according to the Benjamini–Hochberg procedure using a false discovery rate (FDR) of 0.05^28^.

### Reporting Summary

Further information on research design is available in the Nature Research Reporting Summary linked to this article.

## Supplementary information

REPORTING SUMMARY

## Data Availability

The data that support the findings of this study are not openly available due to restrictions (data containing information that could compromise research participant privacy/consent) and are available from the corresponding author (CKL) upon reasonable request.

## References

[1] Fett AK (2011). The relationship between neurocognition and social cognition with functional outcomes in schizophrenia: a meta-analysis. Neurosci.Biobehav.Rev..

[2] Bliksted V, Fagerlund B, Weed E, Frith C, Videbech P (2014). Social cognition and neurocognitive deficits in first-episode schizophrenia. Schizophr. Res..

[3] Sparks A, McDonald S, Lino B, O’Donnell M, Green MJ (2010). Social cognition, empathy and functional outcome in schizophrenia. Schizophr. Res..

[4] Javed A, Charles A (2018). The importance of social cognition in improving functional outcomes in schizophrenia. Front. Psychiatry.

[5] Savla GN, Vella L, Armstrong CC, Penn DL, Twamley EW (2013). Deficits in domains of social cognition in schizophrenia: A meta-analysis of the empirical evidence. Schizophr. Bull.

[6] McDonald S, Flanagan S, Rollins J, Kinch J (2003). TASIT: a new clinical tool for assessing social perception after traumatic brain injury. J Head Trauma Rehabil.

[7] Green MF (2008). Social cognition in schizophrenia: an NIMH workshop on definitions, assessment, and research opportunities. Schizophr. Bull..

[8] Lavoie MA (2013). Social cognition in first-degree relatives of people with schizophrenia: a meta-analysis. Psychiatry Res..

[9] Blokland GAM (2017). Heritability of neuropsychological measures in schizophrenia and nonpsychiatric populations: a systematic review and meta-analysis. Schizophr. Bull..

[10] Toulopoulou T (2007). Substantial genetic overlap between neurocognition and schizophrenia: genetic modeling in twin samples. Arch. Gen. Psychiatry.

[11] Lemvigh C (2020). Heritability of memory functions and related brain volumes: a schizophrenia spectrum study of 214 twins. Schizophr. Bull. Open.

[12] Lemvigh, C. et al. Heritability of specific cognitive functions and associations with schizophrenia spectrum disorders using CANTAB: a nation-wide twin study. *Psychol. Med*. 1–14 10.1017/S0033291720002858 (2020). 10.1017/S0033291720002858 32779562

[13] Besteher B, Brambilla P, Nenadić I (2020). Twin studies of brain structure and cognition in schizophrenia. Neurosci. Biobehav. Rev..

[14] Green MF, Horan WP, Lee J (2015). Social cognition in schizophrenia. Nat. Rev. Neurosci..

[15] Bliksted V, Videbech P, Fagerlund B, Frith C (2017). The effect of positive symptoms on social cognition in first-episode schizophrenia is modified by the presence of negative symptoms. Neuropsychology.

[16] Fiszdon JM, Fanning JR, Johannesen JK, Bell MD (2013). Social cognitive deficits in schizophrenia and their relationship to clinical and functional status. Psychiatry Res..

[17] Chen S, Liu Y, Liu D, Zhang G, Wu X (2021). The difference of social cognitive and neurocognitive performance between patients with schizophrenia at different stages and influencing factors. Schizophr. Res. Cogn..

[18] Mancuso F, Horan WP, Kern RS, Green MF (2011). Social cognition in psychosis: multidimensional structure, clinical correlates, and relationship with functional outcome. Schizophr. Res..

[19] van Dongen J, Slagboom PE, Draisma HHM, Martin NG, Boomsma DI (2012). The continuing value of twin studies in the omics era. Nat. Rev. Genet..

[20] Pinkham AE, Harvey PD, Penn DL (2018). Social cognition psychometric evaluation: results of the final validation study. Schizophr. Bull.

[21] Wing JK (1990). SCAN. Schedules for clinical assessment in neuropsychiatry. Arch. Gen. Psychiatry.

[22] McDonald S (2006). Reliability and validity of the awareness of social inference test (TASIT): a clinical test of social perception. Disabil. Rehabil..

[23] Nelson HE, O’Connell A (1978). Dementia: the estimation of premorbid intelligence levels using the new adult reading test. Cortex.

[24] Wechsler, D. *Manual for the Wechsler Adult Intelligence Scale* 3rd edn (The Psychological Corporation, 1997).

[25] Kay SR, Fiszbein A, Opler LA (1987). The positive and negative syndrome scale (PANSS) for schizophrenia. Schizophr. Bull.

[26] Jones SH, Thornicroft G, Coffey M, Dunn G (1995). A brief mental health outcome scale. Reliability and validity of the global assessment of functioning (GAF). Br. J. Psychiatry.

[27] Fritz CO, Morris PE, Richler JJ (2012). Effect size estimates: current use, calculations, and interpretation. J. Exp. Psychol. Gen..

[28] Benjamini Y, Hochberg Y (1995). Controlling the false discovery rate: a practical and powerful approach to multiple testing. J. R. Stat. Soc..

