# Supplementary material for: A nation-wide twin study of social cognition in schizophrenia spectrum disorders
Source: Schizophrenia (Heidelb). 2022 Mar 2;8(1):12. doi: 10.1038/s41537-022-00223-1 (PMC8891357; doi:10.1038/s41537-022-00223-1)
Supplement: Supplementary file 1 — REPORTING SUMMARY [file 41537_2022_223_MOESM1_ESM.pdf]

nature portfolio

Corresponding author(s): Cecilie Koldbæk Lemvigh

Last updated by author(s): 08-10-2021

Reporting Summary

Nature Portfolio wishes to improve the reproducibility of the work that we publish. This form provides structure for consistency and transparency in reporting. For further information on Nature Portfolio policies, see our Editorial Policies and the Editorial Policy Checklist.

Statistics

For all statistical analyses, confirm that the following items are present in the figure legend, table legend, main text, or Methods section.

n/a	Confirmed	
☐	☒	The exact sample size (n) for each experimental group/condition, given as a discrete number and unit of measurement
☒	☐	A statement on whether measurements were taken from distinct samples or whether the same sample was measured repeatedly
☐	☒	The statistical test(s) used AND whether they are one- or two-sided<br>*Only common tests should be described solely by name; describe more complex techniques in the Methods section.*
☒	☐	A description of all covariates tested
☐	☒	A description of any assumptions or corrections, such as tests of normality and adjustment for multiple comparisons
☐	☒	A full description of the statistical parameters including central tendency (e.g. means) or other basic estimates (e.g. regression coefficient) AND variation (e.g. standard deviation) or associated estimates of uncertainty (e.g. confidence intervals)
☐	☒	For null hypothesis testing, the test statistic (e.g. F, t, r) with confidence intervals, effect sizes, degrees of freedom and P value noted<br>*Give P values as exact values whenever suitable.*
☒	☐	For Bayesian analysis, information on the choice of priors and Markov chain Monte Carlo settings
☒	☐	For hierarchical and complex designs, identification of the appropriate level for tests and full reporting of outcomes
☐	☒	Estimates of effect sizes (e.g. Cohen's d, Pearson's r), indicating how they were calculated

Our web collection on statistics for biologists contains articles on many of the points above.

Software and code

Policy information about availability of computer code

Data collection	no software was used
Data analysis	SPSS version 25, SPSS inc.

For manuscripts utilizing custom algorithms or software that are central to the research but not yet described in published literature, software must be made available to editors and reviewers. We strongly encourage code deposition in a community repository (e.g. GitHub). See the Nature Portfolio guidelines for submitting code & software for further information.

Data

Policy information about availability of data

All manuscripts must include a data availability statement. This statement should provide the following information, where applicable:

- Accession codes, unique identifiers, or web links for publicly available datasets
- A description of any restrictions on data availability
- For clinical datasets or third party data, please ensure that the statement adheres to our policy

The data that support the findings of this study are not openly available due to restrictions (data containing information that could compromise research participant privacy/consent) and are available from the corresponding author (CKL) upon reasonable request.

Field-specific reporting

Please select the one below that is the best fit for your research. If you are not sure, read the appropriate sections before making your selection.

☐ Life sciences ☒ Behavioural & social sciences ☐ Ecological, evolutionary & environmental sciences

For a reference copy of the document with all sections, see nature.com/documents/nr-reporting-summary-flat.pdf

Behavioural & social sciences study design

All studies must disclose on these points even when the disclosure is negative.

Study description	Case-control twin study with patients with schizophrenia spectrum disorders, unaffected co-twins and healthy contrls. Quantative cross-sectional data
Research sample	Representative sample of twins recruited nation-wide in denmark by utilization of the danish registers. Mean age = 40.7, SD = 10.4; 48.6% females.
Sampling strategy	All MZ twin pairs concordant or discordant for a schizophrenia spectrum disorder in Denmark were identified and invited to participate in the clinical study. DZ twins pairs and healthy controls were recruited subsequently matched on age and gender to the included MZ twin pairs.
Data collection	Cognitive data was collected using a mix of computerized and pen-and-paper tasks. Psychopathology and level of functioning were assessed via participants interview. The cognitive data was collected by a trained psychologist and the remaining data was collected by a medical doctor and a research nurse. The research staff was not blided to patient-control status
Timing	Data collection took place from 2011-2017.
Data exclusions	No data were excluded from analyses and were not included in the current analyses.
Non-participation	Two participants only completed questionaires. 9 twins participated without their sibling. Three patients were excluded from the final cohort because their register F2x diagnosis could not be confirmed in the clinical study
Randomization	No randomization

Reporting for specific materials, systems and methods

We require information from authors about some types of materials, experimental systems and methods used in many studies. Here, indicate whether each material, system or method listed is relevant to your study. If you are not sure if a list item applies to your research, read the appropriate section before selecting a response.

Materials & experimental systems

n/a	Involved in the study	
☒	☐	Antibodies
☒	☐	Eukaryotic cell lines
☒	☐	Palaeontology and archaeology
☒	☐	Animals and other organisms
☐	☒	Human research participants
☐	☒	Clinical data
☒	☐	Dual use research of concern

Methods

n/a	Involved in the study	
☒	☐	ChIP-seq
☒	☐	Flow cytometry
☒	☐	MRI-based neuroimaging

Human research participants

Policy information about studies involving human research participants

Population characteristics	See above
Recruitment	All MZ twin pairs concordant or discordant for a schizophrenia spectrum disorder in Denmark were identified and invited to participate in the clinical study. DZ twins pairs and healthy controls were recruited subsequently matched on age and gender to the included MZ twin pairs.
Ethics oversight	The Danish Health and Medicines Authority and The Danish National Committee on Health Research Ethics

Note that full information on the approval of the study protocol must also be provided in the manuscript.

Clinical data

Policy information about clinical studies

All manuscripts should comply with the ICMJE guidelines for publication of clinical research and a completed CONSORT checklist must be included with all submissions.

Clinical trial registration	Not applicable
Study protocol	Not applicable
Data collection	All data were collected at the Mental Health Center Glostrup, Capital Region of Denmark from 2011-2017
Outcomes	This was a large, multimodal study assessing a broad range of cognitive, physiological and brain measures
